# Application of deep-learning–based artificial intelligence in acetabular index measurement

**DOI:** 10.3389/fped.2022.1049575

**Published:** 2023-01-16

**Authors:** Qingjie Wu, Hailong Ma, Jun Sun, Chuanbin Liu, Jihong Fang, Hongtao Xie, Sicheng Zhang

**Affiliations:** ^1^ Department of Pediatric Orthopedics, Anhui Provincial Children’s Hospital, Hefei, China; ^2^ Fifth Clinical Medical College, Anhui Medical University, Hefei, China; ^3^ School of Information Science and Technology, University of Science and Technology of China, Hefei, China

**Keywords:** acetabular index, child, deep learning, artificial intelligence - AI, DDH

## Abstract

**Objective:**

To construct an artificial intelligence system to measure acetabular index and evaluate its accuracy in clinical application.

**Methods:**

A total of 10,219 standard anteroposterior pelvic radiographs were collected retrospectively from April 2014 to December 2018 in our hospital. Of these, 9,219 radiographs were randomly selected to train and verify the system. The remaining 1,000 radiographs were used to compare the system's and the clinicians' measurement results. All plain pelvic films were labeled by an expert committee through PACS system based on a uniform standard to measure acetabular index. Subsequently, eight other clinicians independently measured the acetabular index from 200 randomly selected radiographs from the test radiographs. Bland–Altman test was used for consistency analysis between the system and clinician measurements.

**Results:**

The test set included 1,000 cases (2,000 hips). Compared with the expert committee measurement, the 95% limits of agreement (95% LOA) of the system was −4.02° to 3.45° (bias = −0.27°, *P* < 0.05). The acetabular index measured by the system within all age groups, including normal and abnormal groups, also showed good credibility according to the Bland–Altman principle. Comparison of the measurement evaluations by the system and eight clinicians vs. that of, the expert committee, the 95% LOA of the clinician with the smallest measurement error was −2.76° to 2.56° (bias = −0.10°, *P* = 0.126). The 95% LOA of the system was −0.93° to 2.86° (bias = −0.03°, *P* = 0.647). The 95% LOA of the clinician with the largest measurement error was −3.41° to 4.25° (bias = 0.42°, *P* < 0.05). The measurement error of the system was only greater than that of a senior clinician.

**Conclusion:**

The newly constructed artificial intelligence system could quickly and accurately measure the acetabular index of standard anteroposterior pelvic radiographs. There is good data consistency between the system in measuring standard anteroposterior pelvic radiographs. The accuracy of the system is closer to that of senior clinicians.

## Introduction

Developmental dysplasia of the hip (DDH) is the most common lower extremity deformity in children and the most common skeletal dysplasia causing lower extremity disability, with a prevalence rate of 0.1–2/1,000 (1). At present, it is generally believed that the earlier DDH is treated, the better the prognosis will be. Therefore, early diagnosis of DDH is crucial. The diagnosis of DDH in children >6 months old largely depends on pelvic plain radiography (2). The acetabular index of children can be obtained from plain pelvic radiography, which is a commonly used index for diagnosing DDH and monitoring acetabular development after treatment (3). The acetabular index was measured by the Hilgenreiner method (4, 5). Related studies have shown that there is a large error in the repeatability measurement of acetabular index and the measurement between different doctors (6, 7), which often leads to misdiagnosis and missed diagnosis of DDH. Therefore, it is necessary to construct an artificial system that can accurately and quickly measure the acetabular index.

In recent years, artificial intelligence has been widely applied in the medical field (8). From data collection and image recognition to clinical diagnosis and decision-making, the reliability and superiority of artificial intelligence has been proven to a certain extent (9, 10). In this study, a computerized deep-learning convolutional neural network model was constructed, trained, and validated by using the pelvic plain radiographs labeled by clinicians. Assuming high accuracy in measuring acetabular index, the system can be used to automatically measure acetabular index of plain pelvic films.

## Information and methods

### Pelvic plain film

**Figure 1:**
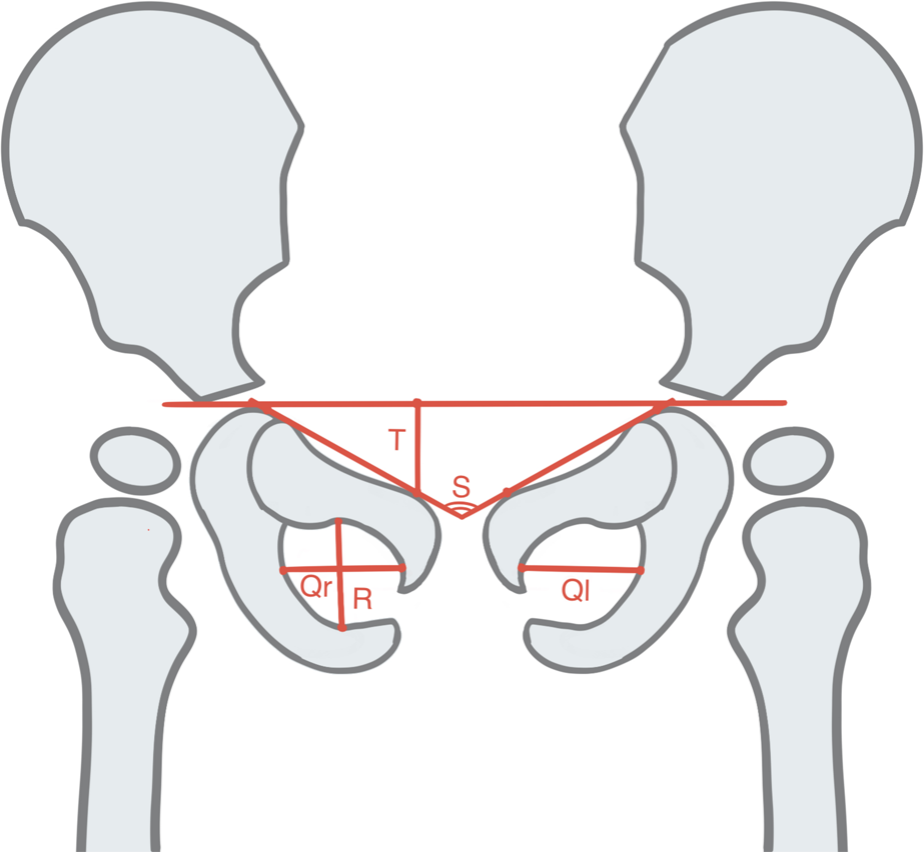
Schematic view of a standard pelvic plain film. Quotient of pelvic rotation (Qr/Ql), Symphysis os-ischium angle (**S**), and Pelvic tilt index (R/T).

**Figure 2:**
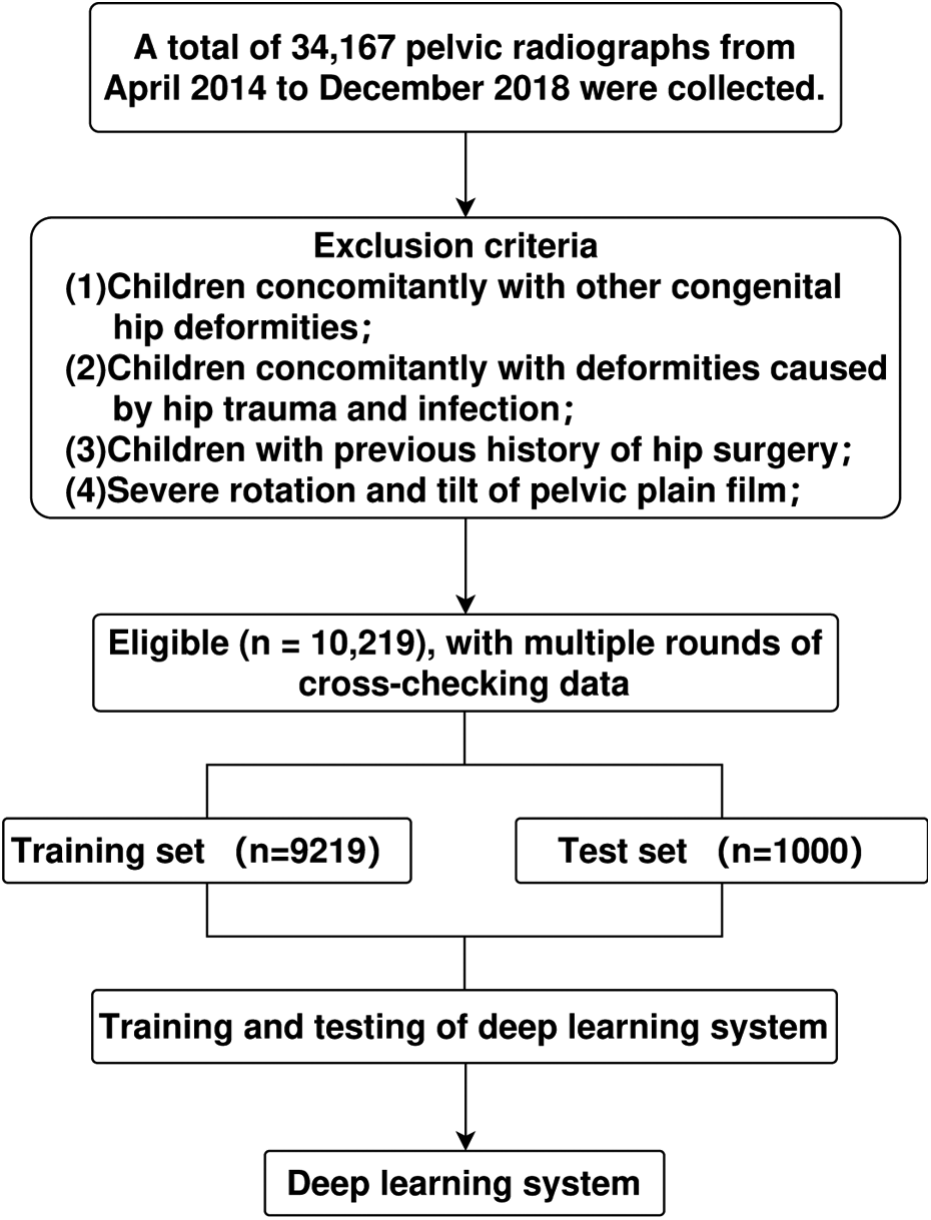
Research flow chart.

This retrospective study was approved by the Medical Research Ethics Committee of the Children's Hospital of Anhui Medical University (approval No.: 20190021). This study collected pelvic radiographs anonymously, so no informed consent was obtained. Standard pelvic plain films from 2014 to 2018 were collected from Radiology Department of our hospital. As shown in Figure 1, a standard pelvic plain film must meet the following three conditions (5, 7): (1) Quotient of pelvic rotation: the ratio of the horizontal diameter of the right obturator foramen to that of the left obturator foramen (Qr and Ql in Figure 1) is between 0.56 and 1.8; (2) Symphysis os-ischium angle: the angle (S in Figure 1) formed by the intersection of two straight lines tangent to the highest point of the ischium and the highest point of pubic symphysis projection ranging from 90 to 135; and (3) Pelvic tilt index: The ratio of the vertical diameter of the obturator foramen to the distance between the superior margin of the pubic bone and the Y-line (“R” and “T” in Figure 1) is between 0.75 and 1.2. The pelvic plain film that does not meet any of the above three conditions is considered to have severe rotation or tilt. Finally, a total of 10,219 anonymous standard pelvic plain films from patients aged between 10 days and 10 years were collected. According to their age, the patients were divided into four subgroups: <6 months, ≥6–12 months, ≥12–24 months and ≥24 months. The hip joints were graded according to the Tönnis criteria (11), where grades 1–4 were considered abnormal, then the hips were divided into a normal group and an abnormal group. Nine thousand two hundred and nineteen of these pelvic radiographs were randomly selected, labeled, and used for training and validating the depth learning system, and the remaining 1,000 pelvic radiographs were used to test the system (Figure 2). The specific distribution of data is depicted in Table 1.

**Table 1:** Clinical characteristics of the study participants.

Characteristic	Entire set (*n *= 10,219)	Training set (*n *= 9,219)	Testing set (*n *= 1,000)
Sex			
Male	2,410	2,232	178
Female	7,809	6,987	822
Age			
Median	1.0	1.0	1.0
0–2 years	8,577	7,663	914
0–6 months	540	468	72
≥ 6–12 months	4,092	3,758	334
≥ 12–24 months	3,945	3,437	508
≥2 years	1,642	1,556	86
Hip			
Normal	9,804	8,838	966
Abnormal	10,634	9,600	1,034

### Expert committee measurements

**Figure 3:**
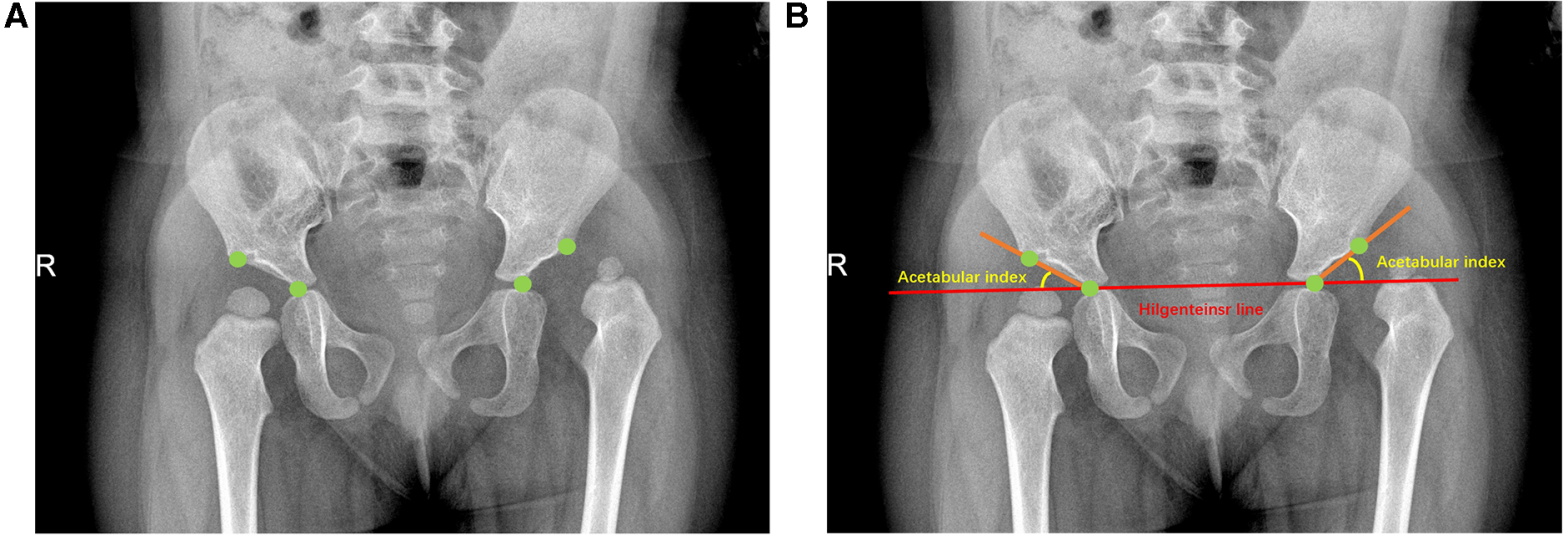
Illustration of standard pelvic plain film labeling. The patient is an 18-month-old child (**A**) The green dots in the figure represent the key labeling points determined according to Hilgenreiner method, namely the Y-type cartilage center point and the acetabular lateral edge point; (**B**) The acetabular index was determined by connecting the key points according to the Hilgenreiner method.

The pelvic plain films used were labeled according to the Hilgenreiner method (Figure 3). Four key points on all images were labeled using a picture archiving and communication system (PACS) workstation (Figure 3A). The line connecting the bilateral acetabular centerpoints was the Hilgenreiner line, and the acetabular index was the angle from the center point of the Y-shaped cartilage to the point on the lateral edge of the acetabulum and the Hilgenreiner line (Figure 3B). In all, 10,219 plain pelvic radiographs were assigned to a panel of 13 clinicians (10 of whom had at least 8 years of clinical experience in pediatric orthopedics; 2 radiologists had more than 15 years of clinical experience in imaging diagnosis; and 1 chief physician had over 25 years of clinical experience in imaging diagnosis of pediatric orthopedics). All the key points on the pelvic plain film were marked according to the unified learning Hilgenreiner method, and the acetabular index value was measured through multiple rounds of cross-examination.

### Network framework

**Figure 4:**
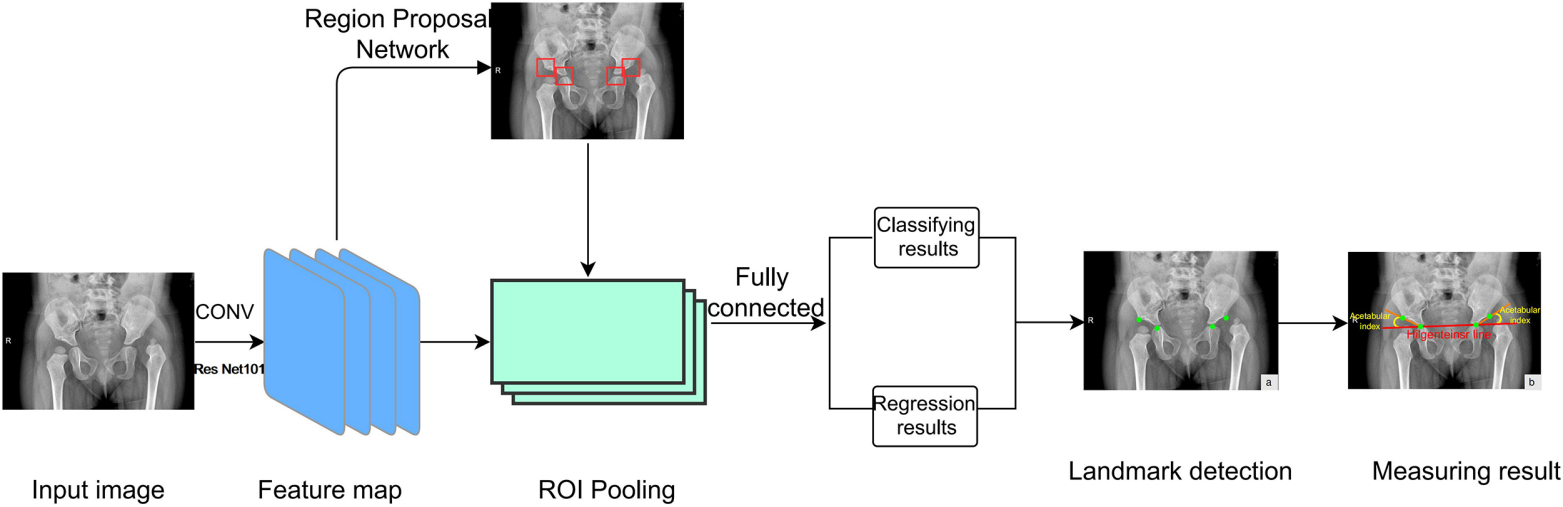
Network framework. The network first uses a series of convolutional layers and ResNet-101 to extract image features. ROI then assembles the neighborhood regions and feature maps generated by RPN. Then, the collected feature regions output two branch results through the full connection layer. Finally, key points are located in these results and measured.

In this study, image automatic recognition and measurement were performed based on a deep-learning method called the Faster RCNN-DDH (FR-DDH) network (Figure 4). For the input image, the network first uses a series of convolution layers and ResNet-101 to obtain the high-dimensional feature map and extract the spatial information. Then, a region proposal network (RPN) generates possible neighborhood regions according to the feature map. Then, the region of interest (ROI) combines the neighborhood region and the feature map. Once assembled, each area is fixed in size. Then, the feature regions with fixed sizes branch the results into two outputs through the full join layer: classification results and regression results. Finally, the key points are located in the corresponding regions, and the measurement results are obtained.

### System test and evaluation

First, the measurement results of the deep learning system and the expert committee on 1,000 pelvic radiographs in the test set were compared and analyzed in this study. Then, 200 plain pelvic radiographs were randomly selected from the test set and assigned to four pediatric orthopedic clinicians (O1, O2, O3, and O4) and four radiologists (R1, R2, R3, and R4) outside the expert committee, where O1 and R1 were senior physicians and the remaining six were junior physicians. The eight physicians independently measured the acetabular index on 200 plain pelvic plates during the same time period; the measurement was not repeated. Finally, the measurement results were compared and analyzed with the measured values of the expert committee. The difference between the measurement results of the 200 pelvic plain films and the lateral values of the expert committee was plotted as a violin figure.

### Data analysis

Acetabular index measurement results were consistent with normal distribution measurement data. All data were statistically analyzed using SPSS 24.0 (IBM Corporation, Armonk, NY, United States); Origin 2021 (Microcal Software Inc, Northampton, MA, United States); and GraphPad Prism 5 (GraphPad Inc, San Diego, CA, United States). Bland–Altman test was used to evaluate the consistency of measurement results of the deep learning system, eight physicians, and the expert committee. Paired *t*-test was used to determine the difference in acetabular index between artificial intelligence and the expert committee. Analysis of variance and two independent samples *t*-test were used to analyze the difference of acetabular index between subgroups. *P* < 0.05 was used to indicate statistically significant differences.

## Results

**Figure 5:**
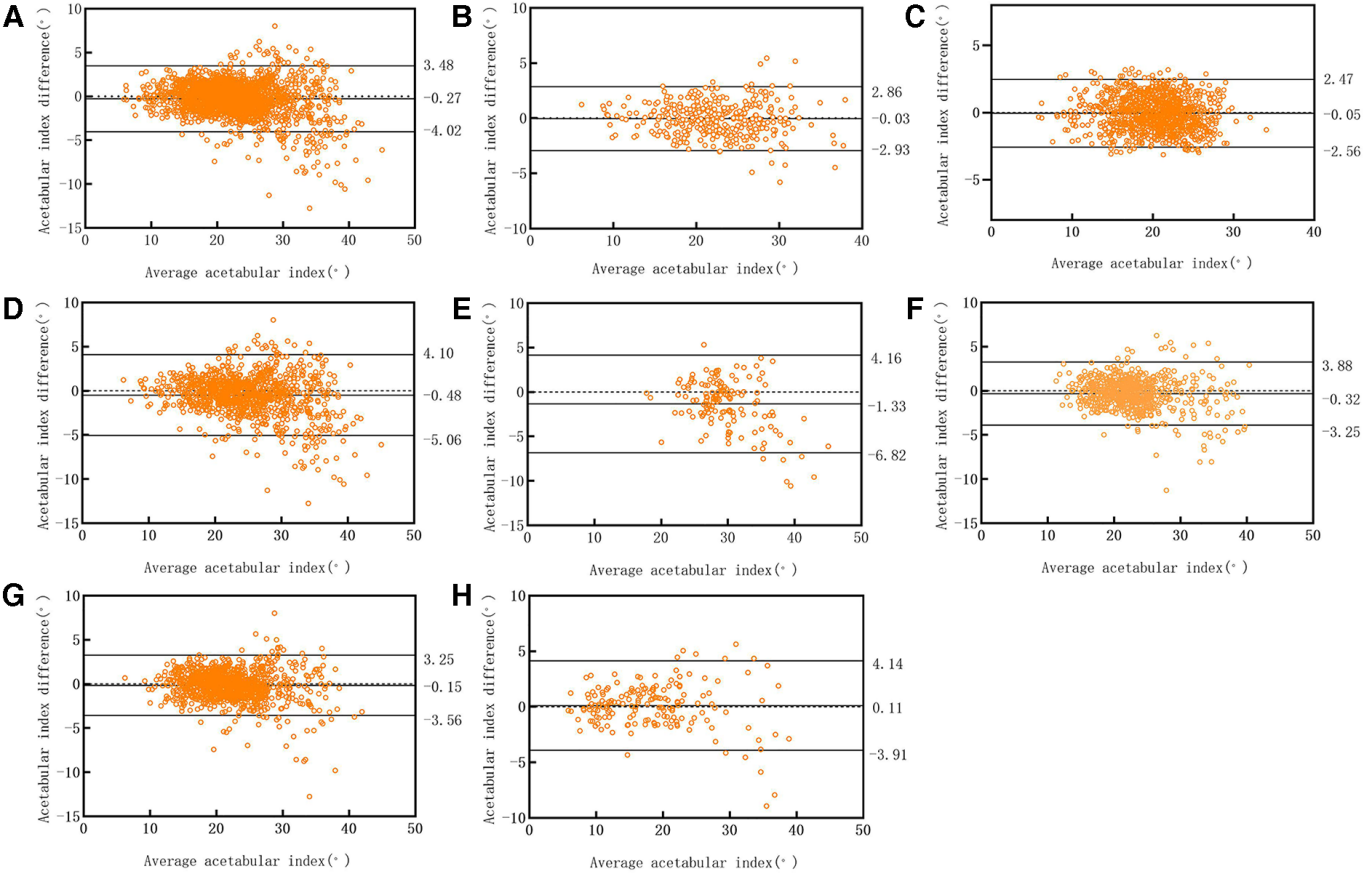
Bland–altman scatter plot: the diagnosis of deep learning system test set was compared to that of the clinician. (**A**) 1,000 cases in the test set; (**B**) 200 cases randomly selected from the test set; (**C**) normal group; (**D**) abnormal group; (**E**) infants <6 months of age; (**F**) infants ≥6–12 months of age; (**G**) infants ≥12–24 months of age; (**H**) children ≥24 months of age.

A total of 10,219 subjects (2,410 male and 7,809 female; mean age: 1.5 ± 1.64; age range: 1 months–10 years) were included in this study. The test set comprised 1,000 patients (190 male and 810 female; mean age: 1.6 ± 1.68 years; age range: 1 months–10 years). Compared to the expert committee measurements, the 95% Limits of Agreement (95% LOA) determined by the Bland–Altman plot for the 1,000 test sets was −4.02° to 3.45° (bias = −0.27°, *P* < 0.05). In the evaluation of 200 pelvic plain films, the deep-learning system measurement showed a 95% LOA of −0.93° to 2.86° (bias = −0.03°, *P* = 0.647) compared to the expert committee measurement. With regards to acetabular index measurements in infants under six months of age, the 95% limit of agreement was −6.82° to 4.16° (bias −1.33°, *P* < 0.05). For infants ≥6–12 months of age, the 95% limit of agreement was −3.25° to 3.88° (bias −0.32°, *P* < 0.05), it was −3.56° to 3.25° (bias −0.15°, *P* ≤ 0.05) for infants ≥12–24 months, while it was −3.91° to 4.14° (bias 0.11°, *P* = 0.468) for children over twenty-four months the 95% limits of agreement, using the Bland–Altman method, for acetabular index measurement in the confirmed normal hip and abnormal hip groups were −2.56° to 2.47° (bias −0.05°, *P* = 0.264) and −5.06° to 4.10° (bias −0.48°, *P* < 0.05), respectively (Figure 5).

In the one-way analysis of variance of acetabular index differences between age groups, only the infants under 6 months showed significant difference compared with other three groups. In the two independent-sample *t*-test revealed significant differences between measured acetabular index differences in the normal hip group and the abnormal hip group.

**Figure 6:**
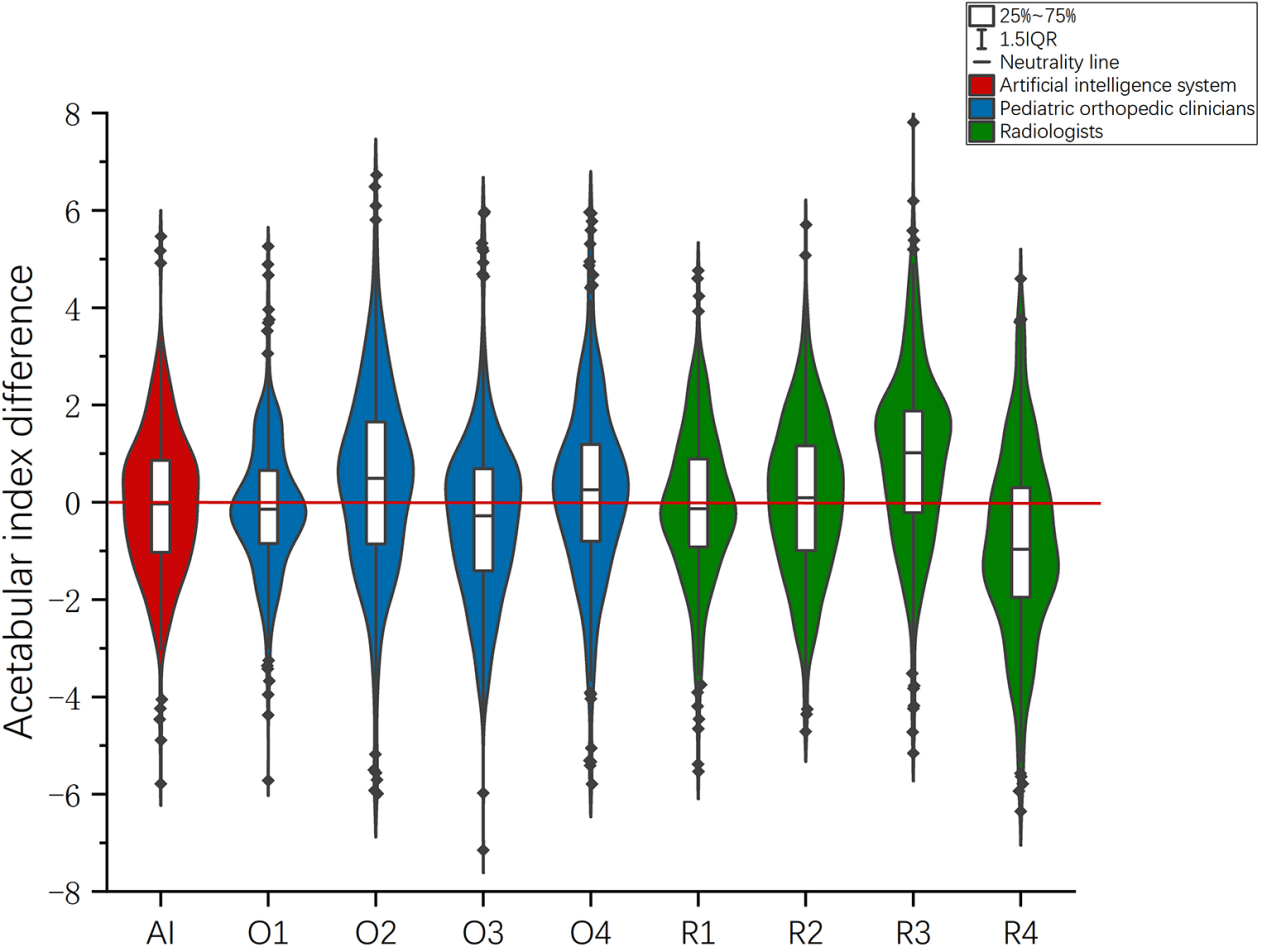
In the evaluation of 200 radiographs, the violin chart was drawn according to the difference between the measurement results and those of the expert committee.

The 95% LOA of senior physician O1 was −2.76° to 2.56° (bias = −0.10°, *P* = 0.126), which was the smallest measurement error among the eight physicians. The 95% LOA in the O2 measurement of junior physicians was −3.41° to 4.25° (bias = −0.42°, *P* < 0.05), which was the largest measurement error among the eight physicians. (Table 2, Figure 6).

**Table 2:** Bland–Altman consistency test results.

	AI	O1	O2	O3	O4	R1	R2	R3	R4
Bias	−0.03	−0.10	0.42	−0.35	0.25	−0.08	0.07	0.92	−0.87
95% LOA									
Lower limit	−2.93	−2.76	−3.41	−3.71	−3.35	−3.11	−3.07	−2.57	−4.57
Upper limit	2.86	2.56	4.25	3.00	3.85	2.95	3.21	4.40	2.83
*P*	0.647	0.126	<0.05	<0.05	<0.05	0.304	0.377	<0.05	<0.05

## Discussion

In this study, we primarily evaluated the ability of artificial intelligence to measure acetabular index. Acetabular index can accurately reflect the development of the acetabulum. It is an important clinical indicator for the diagnosis and efficacy evaluation of DDH. At present, a number of studies have evaluated the application of artificial intelligence in the pediatric musculoskeletal disorders and include predicting scoliosis according to *x*-ray (12), predicting bone age according to hand and wrist *x*-ray (13), determining leglength discrepancy from radiographs (14), quantifying the degree of metopic craniosynostosis from skull CT scans (15), predicting the presence of discoid lateral menisci from radiographs (16).

In this study, we constructed an artificial intelligence system to measure the acetabular index by using 9,219 pediatric standard pelvic radiographs. Comparison of the measurements with those of experienced clinicians demonstrated the reliability of the artificial intelligence tool to accurately measure acetabular indices. In the test set of 1,000 cases (2,000 hips), the 95% LOA for the deep-learning system measurements ranged from −4.02° to 3.45°, when compared to expert committee measurements. However, relevant studies have shown that the measurement error of different clinicians ranges from 3.5° to 10° (6, 7, 17). The results were compared with the clinically allowable error ranges according to the Bland–Altman principle (18). If the 95% LOA is clinically acceptable, the deep learning system is considered reliable to measure the acetabular index.

In this study, cases were divided into a normal hip group and an abnormal hip group according to Tönnis standard. The results showed that the consistency of measured acetabular index in normal group was better than that in abnormal group. This result is due to the fact that in a normal hip *x*-ray, the sclerotic area of the lateral edge of the acetabulum is usually clear and continuous. However, in abnormal hip joints, especially in acetabular dysplasia, the sclerotic area of the lateral edge of the acetabulum is usually unclear and discontinuous, making it difficult to accurately distinguish the lateral edge of the acetabulum on *x*-ray (19). Under these circumstances, measuring the acetabular index may produce large errors. Fortunately, the acetabular index measured by deep learning system in the normal group and the abnormal group, using the Bland–Altman principle, both showed good credibility. In addition, the acetabular index declined more rapidly in the first 3 months of life and then changed more rapidly between the ages of 1 and 5 years (20). Therefore, we divided the cases into four different age groups, and the results showed that the consistency of measured acetabular index in infants under six months of age was worse than that in the other three groups. This is somewhat different from our previous studies (17), and this difference may be due to data collection. We collected cases in infants under six months of age during our retrospective data collection, so we included this in our study.

The acetabular index measured by different physicians is marginally different, which may lead to delayed diagnosis or excessive treatment for children with DDH. Relevant literature has pointed out that it is necessary to compare clinicians' accuracy with that of artificial intelligence-based tools (21). In the evaluation of the measurement results of the deep learning system and the eight clinicians, the measurement error of the most senior physician O1 (95% LOA: −2.76° to 2.56°) was the smallest, followed by the deep learning system (95% LOA: −0.93° to 2.86°) and the senior radiologist R1 (95% LOA: −3.11° to 2.95°) (Table 2). The measurement errors of the remaining six junior physicians were greater than those of the artificial intelligence system, and the physician with the largest measurement error was O2 (95% LOA: −3.41° to 4.25°). This means that the accuracy of the deep learning system in measuring the acetabular index is second only to that of the senior orthopedist O1. Therefore, it can be concluded that the measurement reliability of the deep learning system is high whether compared with the pediatric orthopedist or the radiologist. However, the primary doctor may have insufficient experience, so the error is slightly larger. In the violin figure of acetabular index difference, it can also be seen that the median value and overall distribution of the difference between the measured values of the deep learning system and the measured values of the expert committee are close to 0. Therefore, it can be considered that the deep learning system is a reliable tool to measure acetabular index, and the measurement accuracy may be improved to a certain extent by using the deep learning system-based measurement as a reference for clinicians when reading films. In addition, the time required for the deep learning system to identify and measure a flat pelvic slice is about 1 s, which greatly improves the efficiency of slice reading.

This study has some limitations. First, the acetabular index measured by the Committee of Experts was obtained through multiple rounds of cross-examination by multiple authoritative physicians. However, there are still some differences when compared with the true value of acetabular index. Second, previous studies have shown that the deep learning system is different for measuring the pelvic plain films of dislocated and non-dislocated, older and younger children (17). In this study, the degree of dislocation and age were not included in the assessment. Therefore, there is a certain bias in the result analysis of the 200 randomly selected plain pelvic radiographs. Moreover, the deep learning system in this study cannot automatically identify the rotation and tilt of the image, nor can it automatically correct the errors caused by the tilt rotation. Finally, this is a single center study. Therefore, in the future we aim to collect more data and further optimize the system algorithm to reduce errors. More multicenter comparative studies are currently being conducted to improve the accuracy of the system measurements.

In summary, a deep-learning–based artificial intelligence system that can automatically measure acetabular index was successfully constructed in this study. The results of the artificial intelligence system compared with clinicians also confirmed the reliability of the system in measuring acetabular index. At the same time, the system greatly improved the efficiency of film reading and reduced human errors in the measurement. In the future, more studies are needed to further evaluate and optimize the artificial intelligence system, and strive for its early and wide application in clinical practice.

## Data Availability

The original contributions presented in the study are included in the article/Supplementary Material, further inquiries can be directed to the corresponding author.

## References

[1] Gokharman FD Aydin S Fatihoglu E Ergun E Kosar PN. Optimizing the time for developmental dysplasia of the hip screening: earlier or later? Ultrasound Q. (2019) 35(2):130–5. 10.1097/ruq.0000000000000348 29509577

[2] Zhang CY Chen BC Wang ZG Cai HQ Yang J Li YC Study on the cohesion between ultrasound and x-ray examination of hip joint in children. Chin J Clin. (2012) 6(24):8372–3. 10.3877/cma.j.issn.1674-0785.2012.24.144

[3] Yang S Zusman N Lieberman E Goldstein RY. Developmental dysplasia of the hip. Pediatr. (2019) 143(1):e20181147. 10.1542/peds.2018-1147 30587534

[4] Thieme WT Thiersch JB. Classic translation: hilgenreiner on congenital hip dislocation. J Pediatr Orthop. (1986) 6(2):202–14. 10.1097/01241398-198603000-00016 3514668

[5] Tönnis D. Normal values of the hip joint for the evaluation of x-rays in children and adults. Clin Orthop Related Res. (1976) 119(119):39–47. 10.1097/00003086-197609000-00007 954321

[6] Spatz DK Reiger M Klaumann M Miller F Stanton RP Lipton GE. Measurement of acetabular index intraobserver and interobserver variation. J Pediatr Orthop. (1997) 17(2):174–5. 10.1097/00004694-199703000-00007 9075091

[7] Boniforti FG Fujii G Angliss RD Benson MK. The reliability of measurements of pelvic radiographs in infants. J Bone Joint Surg. (1997) 79(4):570–5. 10.1302/0301-620x.79b4.7238 9250741

[8] Ramkumar PN Kunze KN Haeberle HS Karnuta JM Luu BC Nwachukwu BU Clinical and research medical applications of artificial intelligence. Arthroscopy. (2021) 37(5):1694–7. 10.1016/j.arthro.2020.08.009 32828936 PMC7441013

[9] Chan HP Samala RK Hadjiiski LM Zhou C. Deep learning in medical image analysis. Adv Exper Med Biol. (2020) 1213:3–21. 10.1007/978-3-030-33128-3_1 32030660 PMC7442218

[10] Li Q Liu WY. Applications and future prospects of artificial intelligence in pediatric surgery. Chin J Pediatr Surg. (2021) 42(1):76–81. 10.3760/cma.j.cn421158-20190813-00496

[11] Tönnis D. General Radiography of the Hip Joint. Springer Berlin Heidelberg, (1987).

[12] García-Cano E Arámbula Cosío F Duong L Bellefleur C Roy-Beaudry M Joncas J. Prediction of spinal curve progression in adolescent idiopathic scoliosis using random forest regression. Comput Biol Med. (2018) 103:34–43. 10.1016/j.compbiomed.2018.09.029 30336363

[13] Zhang L Chen J Hou L Xu Y Liu Z Huang S Clinical application of artificial intelligence in longitudinal image analysis of bone age among GHD patients. Front Pediatr. (2022) 10:986500. 10.3389/fped.2022.986500 36440334 PMC9691878

[14] Zheng Q Shellikeri S Huang H Hwang M Sze RW. Deep learning measurement of leg length discrepancy in children based on radiographs. Radiol. (2020) 296(1):152–8. 10.1148/radiol.2020192003 32315267

[15] Bhalodia R Dvoracek LA Ayyash AM Kavan L Whitaker R Goldstein JA. Quantifying the severity of metopic craniosynostosis: a pilot study application of machine learning in craniofacial surgery. J Craniofac Surg. (2020) 31(3):697–701. 10.1097/SCS.0000000000006215 32011542 PMC7202995

[16] Ha CW Kim SH Lee DH Kim H Park YB. Predictive validity of radiographic signs of complete discoid lateral meniscus in children using machine learning techniques. J Orthop Res. (2020) 38(6):1279–88. 10.1002/jor.24578 31883134

[17] Zhang SC Sun J Liu CB Fang JH Xie HT Ning B. Clinical application of artificial intelligence-assisted diagnosis using anteroposterior pelvic radiographs in children with developmental dysplasia of the hip. Bone Joint J. (2020) 102-b(11):1574–81. 10.1302/0301-620x.102b11.Bjj-2020-0712.R2 33135455

[18] Bland JM Altman DG. Statistical methods for assessing agreement between two methods of clinical measurement. Lancet. (1986) 1(8476):307–10. 10.1016/S0140-6736(86)90837-8 2868172

[19] Kim HT Kim JI Yoo CI. Diagnosing childhood acetabular dysplasia using the lateral margin of the sourcil. J Pediatr Orthop. (2000) 20(6):709–17. 10.1097/00004694-200011000-00003 11097241

[20] Kuong EE Gardner WT Koljonen PA Mahapatra SK Chow W. Normal radiographic parameters in paediatric pelvic radiographs from a Chinese population. J Pediatr Orthop B. (2017) 26(4):336–9. 10.1097/BPB.0000000000000426 28079743

[21] Offiah AC. Current and emerging artificial intelligence applications for pediatric musculoskeletal radiology. Pediatr Radiol. (2021). 10.1007/s00247-021-05130-8 PMC9537230 34272573

